# Editorial: Current perspectives on the role of lipids in allergic reaction

**DOI:** 10.3389/fmolb.2024.1364003

**Published:** 2024-01-15

**Authors:** Cerrone Cabanos, Ekaterina Finkina

**Affiliations:** ^1^ Laboratory of Food Quality Design and Development, Graduate School of Agriculture, Kyoto University, Uji, Kyoto, Japan; ^2^ Science-Educational Center, M. M. Shemyakin and Yu A. Ovchinnikov Institute of Bioorganic Chemistry, The Russian Academy of Sciences, Moscow, Russia

**Keywords:** allergens, hydrophobic, lipid, protein-lipid interactions, toll like receptors (TLR), allergic sensitization

In many regions of the world, allergies are now the most common chronic disorder, impacting up to 40% of the population globally (Prescott et al., 2013). Lipids are nonpolar, hydrophobic, or amphipathic small molecules that can be bound or delivered with allergenic proteins to the innate immune system. It has now been demonstrated that these lipid cargos or ligands may entirely or partially influence these allergic reactions. In this Frontiers Research Topic, we present papers that showcase the current breadth of lipids and lipid-conjugates that are associated with allergens, influence allergic sensitization, and our current understanding of their molecular mechanisms.

In their systematic review, Hopkins et al. produced compelling evidence for the function of lipids in allergen sensitization based on current literature. Through various mechanisms, intrinsic lipids derived from allergen sources can interact with proteinaceous allergens to both promote and decrease allergic sensitization. The proposed mechanisms include lipids modifying the structure of allergenic proteins to prevent breakdown in the gut, decreasing the uptake of allergenic proteins by the dendritic cells to suppress immune tolerance, controlling Th2 cytokines, triggering invariant natural killer T cells via CD1d-mediated presentation as well as directly interacting with dendritic cells, toll-like receptors, keratinocytes, and epithelial cells.

Guryanova et al. also reviewed the sensitization mechanism of pollen allergens, a major cause of asthma and allergic rhinitis. Plant pollen contains a number of panallergens, which are allergens widely distributed in nature. They investigated the characteristics of these allergens as well as how they interacted with lipids and other hydrophobic compounds contributing to pollen sensitivity. These proteins were classified as belonging to the classes of LTPs, Bet v1 homologs, profilins, beta-expansins, polcalcins, and the Group-5 allergens. They have also demonstrated that allergic potency of protein allergens is determined not only by their innate immune-modulating qualities and structural and physicochemical characteristics but also by their capacity to bind hydrophobic ligands that may influence their allergenic qualities. Moreover, pollen microorganisms and plant lipids may contribute significantly to sensitivity to allergens that bind to lipids as well as those that do not because of their adjuvant qualities. They also reviewed how direct stimulation of antigen-presenting cells and indirect activation of innate lymphoid cells by respiratory and intestinal epithelial cells is a crucial factor in allergic inflammation that results in sensitization.

Further, it was demonstrated by Palladino et al. that peanut lipids affect the way bronchial epithelial cells (BEC) react to the peanut allergens Ara h 1 and Ara h 2 through reducing barrier permeability. They have shown that BEC line 16HBE14o-polarized monolayers were apically activated by peanut allergens and/or their associated lipids. They have demonstrated that Ara h 1 and Ara h 2 penetrated the epithelial barrier and had an effect on the barrier integrity of these BECs. They have also reported that pro-inflammatory mediators were released under the influence of Ara h 1. These lipids reduced the quantity of allergens that passed through the epithelial layer, decreased paracellular permeability, and enhanced the barrier function of the BEC monolayers. The translocation of Ara h 1 and Ara h 2 across the airway epithelium, the generation of a pro-inflammatory reaction, and the identification of a significant role for peanut lipids in regulating the quantity of allergens that are able to pass the epithelial barrier were all demonstrated by this work.

While some lipids cause sensitization and allergic inflammation, others cause anti-inflammation. Mead acid, an oleic acid metabolite, has been shown by Saika et al. to prevent retinol-induced irritant contact dermatitis (ICD). Although retinol and its derivatives are frequently used as an additive in topical skincare products to cure acne and wrinkles and slow down the skin aging process, they can also have unfavorable side effects, such as ICD. They previously demonstrated that by preventing neutrophil infiltration and leukotriene B4 generation by neutrophils, mead acid (5,8,11-eicosatrienoic acid) reduced skin inflammation in allergic contact hypersensitivity induced by dinitrofluorobenzene. In their recent work, they have uncovered using a mouse model that mead acid prevented keratinocyte abnormalities such as hyperproliferation. Mead acid consistently decreased p38 mitogen-activated protein kinase phosphorylation, which is an important signaling route in retinol-induced keratinocyte hyperplasia. Mead acid’s inhibitory effects were related to the suppression of both hyperproliferation of the keratinocyte and the gene activation of neutrophil chemo-attractants, including Cxcl1 and Cxcl2, and were enabled via a peroxisome proliferator-activated receptor-α-pathway.

Wenger et al. presented a review of the function of Toll-like receptors (TLR) in the management of allergic disorders. The ligands of toll-like receptors, which serve as sensors connecting the innate and adaptive immune responses include lipids, glycoproteins, lipoproteins, and nucleic acids, among other substances originating from foreign microorganisms. Not only are genetic differences in TLR-related genes linked to the pathophysiology of allergic disorders, such as allergic rhinitis and asthma, but allergic and non-allergic people exhibit these Research Topic differently. The elucidation of TLRs role in immunoglobulin E-mediated disorders remains a massive challenge because of the intricate interactions between genes, the environment, and allergen sources that bind to or associate with lipids. The review also covered the expression of TLRs in immune system organs and cell types involved in allergy, their role in regulating immune responses associated with or protective against allergies, and how different environmental factors, like exposure to bacteria, viruses, or air pollutants, activate TLRs differently, leading to the development of allergies. Their work also focused on the interaction between allergen sources and TLRs and how targeting TLRs could be used in novel therapeutic strategies.

In conclusion, these scientific works cover different aspects of lipid-associated allergen sensitization and immune responses, showing interesting results that will help to understand the role of lipids in allergic disorders better. Taken together, these findings can aid in the development of innovative approaches to treat or minimize allergen sensitization.
